# Knowledge, attitude and practice of women in Campinas, São Paulo, Brazil with respect to physical exercise in pregnancy: a descriptive study

**DOI:** 10.1186/1742-4755-8-31

**Published:** 2011-11-03

**Authors:** Carmen P Ribeiro, Helaine Milanez

**Affiliations:** 1 Department of Obstetrics and Gynecology, School of Medical Sciences, University of Campinas, (UNICAMP), São Paulo, Brazil

## Abstract

**Background:**

Pregnancy is a good time to develop healthy lifestyle habits including regular exercise and good nutrition. Programs of physical exercise for pregnant women have been recommended; however, there are few references on this subject in the literature. The objective of this study was to evaluate the knowledge, attitude and practice of pregnant women with respect to appropriate physical exercise during pregnancy, and also to investigate why some women do not exercise during pregnancy.

**Methods:**

A descriptive study was conducted in which 161 women of 18 to 45 years of age were interviewed in the third trimester of pregnancy. These women were receiving prenatal care at National Health Service (SUS) primary healthcare units and had no pathologies for which physical exercise would constitute a risk. The women were selected at an ultrasonography clinic accredited to the SUS in Campinas, São Paulo. A previously elaborated knowledge, attitude and practice (KAP) questionnaire was used to collect data, which were then stored in an Epinfo database. Statistical analysis was conducted using Pearson's chi-square test and Fisher's exact test to evaluate the association between the study variables (p < 0.05).

**Results:**

Almost two-thirds (65.6%) of the women were sufficiently informed about the practice of physical exercise during pregnancy and the vast majority (93.8%) was in favor of it. Nevertheless, only just over 20% of the women in this sample exercised adequately. Significant associations were found between an adequate knowledge of physical exercise during pregnancy and education level (p = 0.0014) and between the adequate practice of physical exercise during pregnancy and having had fewer pregnancies (p = 0.0001). Lack of time and feeling tired and uncomfortable were the principal reasons given by the women for not exercising.

**Conclusion:**

These results suggest that women's knowledge concerning the practice of physical exercise during pregnancy is reasonable and their attitude is favorable; however, relatively few actually exercise during pregnancy.

## Background

Attitudes toward exercise during pregnancy have changed dramatically over the past 20 years. Recent studies show that, in most cases, exercise is safe for both the mother and fetus during pregnancy, and support the recommendation to initiate or continue exercise in most pregnancies [1,2]. The greater the number of pregnant women who want to participate in sports activities, the more important becomes the question about influence of exercise on mother and fetus [3].

Pregnancy is a good time to develop healthy lifestyle habits including regular exercise [1]. Physical exercise programs for pregnant women are recommended and are steadily increasing in popularity [4]. The benefits included maintenance of prenatal aerobic and musculoskeletal fitness levels, prevention of excessive maternal weight gain, facilitation of labor, help for gestational glucose control and improve psychological adjustment to changes of pregnancy [1].

With the objective of providing information for physical exercise programs for pregnancy women, the American College of Obstetricians and Gynecologists (ACOG) published recommendations for the safe practice of water or ground-based physical activities during pregnancy. In accordance with these recommendations, irrespective of the pregnant woman's physical fitness level, exercise should be low-impact, moderate-intensity and regular. Sedentary women should increase their activities gradually and progressively [2].

The prevalence of physical activity is low among Brazilian pregnant women (12, 9%). Although physical activity is not perceived as being pregnancy- threatening, and current guidelines recommend it, this population's behavior does not seem to changing [5].

Some authors have investigated the beliefs and attitudes of women with respect to the practice of physical activity in pregnancy [6-10] and the factors that affect their behavior in relation to exercise [6]. The objective of the present study was to evaluate the knowledge, attitudes and practice of pregnant women in relation to physical exercise during pregnancy and to evaluate why some women fail to exercise.

## Methods

A descriptive study was conducted in Campinas, a city of approximately 1.5 million inhabitants in the state of São Paulo, Brazil where social development level is good. More than 70% of the population depends on the free healthcare provided by the Brazilian National Health Service (SUS). Between March and August 2009, 161 women who fulfilled the following inclusion criteria were interviewed: age 18-45 years, gestational age ≥ 28 weeks, receiving prenatal care at the primary healthcare units affiliated with the SUS in the city, and having no pathologies that would render physical exercise risky in accordance with ACOG recommendations [2]. Women with any physical deficiency that restricted their ability to perform any type of physical exercise were excluded from the study. The participants were selected at an ultrasonography clinic accredited to the SUS. Women waiting for their scan were approached and asked if they would agree to participate in the study. All women fulfilled the inclusion criteria agreed to participate and were then interviewed individually.

Sample size was calculated for the proportion of women who consider the practice of physical exercise during pregnancy to be beneficial (p = 95%) and for the proportion of women who exercise during pregnancy (p = 71%) [6]. For all calculations, a significance level of 5% was adopted. Assuming a sample error of 7%, sample size was defined as 161 women.

A structured knowledge, attitude and practice (KAP) questionnaire [11] containing pre-coded and open questions was used for data collection. The questionnaire requested information on the women's general characteristics, their knowledge concerning physical exercise during pregnancy, the need to exercise, exercise during pregnancy and barriers to exercising during pregnancy. The questionnaire was pre-tested in a similar population sample to that included in the study.

For data analysis, the following definitions were adopted:

### Knowledge

Notions about physical exercise during pregnancy were classified as ***adequate ***(had heard about it and knew what kinds of exercise are indicated and which should not be performed; in accordance with the ACOG definition [2], appropriate exercises for pregnant women are low-impact, moderate-intensity activities such as walking, water aerobics, stretching, yoga and swimming, while high-impact forms of exercise such as strength training and running should not be performed); or ***inadequate ***(had heard about physical exercise during pregnancy but did not know what kind of exercise is indicated or contraindicated, or had never heard of physical exercise during pregnancy).

### Attitude

The opinion of the woman interviewed with respect to performing physical exercise during pregnancy was classified as ***favorable ***or ***unfavorable***.

### Practice

The study investigated whether in accordance with ACOG recommendations [2], and as reported by the woman herself, the participant routinely practiced a form of physical exercise specifically developed for pregnant women, three times a week during the current pregnancy. Practice was classified as ***adequate ***(exercised at least three times a week in accordance with ACOG recommendations); or ***inadequate ***(did not exercise during pregnancy or exercised less than three times a week or performed exercise that is inappropriate).

The association was evaluated between adequate knowledge, attitude and practice of physical exercise in pregnancy and some sociodemographic and obstetric characteristics such as age, education level, parity, paid employment and income.

The SAS statistical software package, version 9.2, was used to perform univariate and bivariate data analyses using the chi-square test or Fisher's exact test, as required. The study protocol was approved by the Internal Review Board (IRB) of the Center for Women's Integrated Healthcare (CAISM), Department of Obstetrics and Gynecology, University of Campinas (UNICAMP) and by the IRB of the School of Medical Sciences of the same institution (approval letter #068/2008). All the participants signed an informed consent form prior to their interview.

## Results

The sociodemographic and obstetric characteristics of the 161 women interviewed are shown in Table 1. The mean age of the participants was 25 years and mean gestational age at the time of the interview was 32 weeks. Slightly more than a quarter of the women (27.3%) described themselves as white. Almost two-thirds were in non-stable union and two-thirds had been pregnant more than once. Slightly more than half (53.4%) reported having at least some high school education. Only a third (31.7%) were in paid employment and mean personal income was US$397.00.

**Table 1:** Distribution of the women in accordance with their sociodemographic and obstetric characteristics.

Variables	n	%
***Age ***(years)		
18-20	18	11.2
20-29	108	67.1
≥ 30	35	21.7
***Gestational age ***(weeks)		
28-32	86	53.8
33-36	52	32.5
> 36	22	13.7
Data missing	1	0.6
***Skin color*** White Non-white	44 117	27.3 72.7
***Marital status*** Stable union Not in stable union	47 114	29.2 70.8
***Number of pregnancies*** 1 ≥ 2	57 104	35.4 64.6
***Schooling***		
Primary education	72	44.8
High school	86	53.4
University	3	1.8
***Paid employment*** Yes No Data missing	51 107 3	31.7 66.5 1.8
***Income ***(minimum salaries)		
≤ 1	7	13.7
> 1-3 > 3	42 2	82.4 3.9

As shown in Table 2, 68.1% of the women interviewed stated that they had heard about the performance of physical exercise during pregnancy and in 65.6% of cases this knowledge was classified as adequate, based on the established criteria. The principal sources of information on physical exercise mentioned by the women were: television, books/magazines and healthcare units.

**Table 2:** Sources and adequacy of knowledge on the practice of physical exercise during pregnancy.

		n	%
Had heard about physical exercise*	Yes	109	68.1
	No	51	31.9
Knowledge is adequate **	Yes	103	65.6
	No	54	34.4
Sources of information ***	Television	89	55.3
	Books and magazines	39	24.2
	Healthcare units	33	20.5
	Friends	18	11.2
	Doctors	15	9.3
	Internet	11	6.8

* Data missing for 1 woman.

** Data missing for 4 women.

*** More than one response was permitted.

In the great majority of cases (93.8%), the attitude of the women towards physical exercise in pregnancy was classified as adequate. The main reasons given for considering that this type of exercise is necessary were: to make childbirth easier, to improve the health of the mother and baby, to ease pain and discomfort, promote well-being and to avoid putting on too much weight (Table 3).

**Table 3:** Attitude of the women towards the need to exercise during pregnancy and the reasons for doing so.

	n	%
***Attitude***		
Adequate	151	93.8
Inadequate	10	6.2
***Reason why physical exercise during pregnancy is necessary***		
Makes delivery easier/improves maternal and fetal health	94	58.4
Relieves pain, discomfort; improves well-being	44	27.3
Avoids weight gain	13	8.1

Less than a third of the women (29%) stated that they had exercised or were exercising during their current pregnancy; however, this practice was classified as adequate, i.e. occurring at least three times a week, in only slightly more than 20% of the women. Walking being the most accomplished. The principal barriers to exercising reported by this group of women were lack of time and feeling too tired and uncomfortable (Table 4).

**Table 4:** Practice and adequacy of physical exercise during pregnancy and the barriers to exercising, as reported by the women interviewed.

		n	%
***Has exercised or currently exercises during this pregnancy***	Yes	48	29.0
	No	113	71.0
***Is exercise adequate?***	Yes	37	22.9
	No	124	77.1
Barriers to the practice of physical exercise* (n = 113)			
	Lack of time	63	55.8
	Feels very tired	21	18.6
	Feels uncomfortable	16	14.2
	Does not like exercising	14	12.4
	Lack of information	8	7.1
	Is afraid that it will be harmful	5	3.1

* Difference in the total number of women may reflect more than one response.

Adequate knowledge about physical exercise during pregnancy was associated with better schooling, while the adequate practice of physical exercise was associated with nulliparity (Table 5).

**Table 5:** Knowledge, attitude and practice of the women with respect to physical exercise in pregnancy, according to selected characteristics.

	Knowledge			Attitude			Practice		
	**n**	**%**	**p**	**n**	**%**	**p**	**n**	**%**	**p**

***Age ***(years)									
< 25	62	66.0		88	92.6		25	26.3	
> 25	41	65.1	0.91	63	95.5	0.53	12	18.2	0.23
***Schooling***									
Primary	37	52.9		65	90.3		11	15.3	
High school	65	77.4		83	96.5		26	30.2	
University	1	33.3	**0.001**	3	100.0	0.33	0	0	0.45
***Number of Pregnancies***									
1^st ^pregnancy	38	67.9		55	96.5		23	40.4	**0.0001**
2^nd ^pregnancy	37	72.6		48	92.8		8	15.4	
≥ 3 pregnancies	13	48.2	0.09	26	92.9	0.64	2	7.1	
***Paid employment***									
No	67	64.4		100	93.5		24	22.4	
Yes	36	72.0	0.35	48	94.1	1	13	25.5	0.07
***Income***									
≤ 1 MS	4	57.1		8	65.7		1	14.3	
> 1-3 MS	30	79.6		40	95.2		11	26.2	
> 3 MS	2	100.0	0.54	2	100.0	0.44	1	50.0	0.51

MS: Minimum salary.

## Discussion

The women in this study sample were shown to be adequately knowledgeable concerning the practice of physical exercise in pregnancy and their attitude towards exercising was favorable; however, few actually exercised.

The sample population consisted of young women (mean age 25 years) with a mean parity of two. More than half had at least some high school education. These characteristics are similar to those found in the samples described by Pereira et al. [12] and Evenson et al. [13], who evaluated predictors of physical activity during and following pregnancy and barriers to the performance of exercise in white women, Latin American women and women of African descent.

In this study, evaluation of the knowledge, attitude and practice of these women with respect to physical exercise during pregnancy showed findings that are similar to results reported in various other countries [6,7,14-21]. A study that evaluated factors associated with women's perceptions of the safety of physical activity in pregnancy found that women perceive physical exercise as beneficial because they believe it helps control blood glucose levels, minimizes weight gain, improves energy efficiency and mood, makes childbirth easier and contributes towards fetal health [9]. Nevertheless, although these women recognized these advantages, they believed that it was more important to rest and relax during pregnancy than to exercise.

These findings are in agreement with the results of the present study, since although knowledge was satisfactory and the women's attitude was favorable, only a small percentage of the women in this sample exercised during pregnancy, highlighting the fact that practice was inadequate. These findings regarding practice are in agreement with other studies conducted in Brazil such as that carried out in Pelotas, Rio Grande do Sul [5] in which the prevalence of physical activity among pregnant women was low and a study conducted in Campina Grande, Paraíba [22] in which the level of physical activity in pregnant women was inadequate right from the beginning of pregnancy, becoming more so in the final trimester. The present findings are also in agreement with studies from other countries, reporting that few women participate in physical activities during pregnancy [5,12,23-25].

The present study showed that knowledge of physical exercise in pregnancy was significantly higher among the women with better schooling, a finding that was to be expected. Nevertheless, no statistically significant association was found between the practice of exercise and education level or being in paid employment. The practice of physical activity during pregnancy was, however, significantly higher among women who had had fewer pregnancies, nulliparas being the group of women who were most likely to exercise. This may be due to not having to be worried or concerned with other children, hence having more time to carry out other activities including the regular practice of physical exercise. These findings are similar to results reported from a study conducted in Portugal [8].

The set of data presented in studies conducted in Brazil and abroad suggests that, although women are aware of the benefits of physical exercise during pregnancy, they do not behave in accordance with this knowledge, compliance with exercising being low, a fact that was also confirmed in the present study.

As shown in this study, other than tiredness and discomfort, the main reason given by the women for not exercising was lack of time. Other authors have also reported similar findings [9,10,12,13,26]. Some authors suggest that discussing the benefits of exercise during prenatal care and making exercise methods available to ensure that the women who exercise will feel comfortable and safe may stimulate the performance of physical activity during pregnancy.

The fact that the principal barriers to exercising described by the pregnant women in the present study were lack of time and feeling tired and uncomfortable may suggest that many women do not feel motivated to exercise despite being aware of the possible benefits that physical exercise could offer to their health and the health of their baby. Improving guidelines and the counseling given to pregnant women to provide them with solid advice in accordance with the level and type of physical exercise that they would like to perform could have a relative impact on their health, well-being and self-esteem during pregnancy. Despite financial restrictions and time limitations, women receiving prenatal care at primary healthcare units could be encouraged to practice regular physical exercise such as that provided in programs that are already available for the treatment of specific pathologies such as diabetes and hypertension. The "Walking for Health" program has already been implemented at basic healthcare level under decree #79(2008) with interesting results in terms of glycemic control in the population participating in these exercise programs [27].

Studies should be conducted to analyze whether these easily implemented measures could have a positive impact on pregnancy and on perinatal outcome. It is known that certain other factors also prevent women in low-income segments of the population from exercising regularly during pregnancy, specifically factors involving suitable places in which to exercise, safety and adequate supervision. A simple walk under the supervision of a healthcare agent adequately trained and informed by a physical education instructor could represent an initial measure that would be simple to implement and could have a positive effect on these women's health.

The data from the present study refer to a small sample population, since the study is restricted to only 161 pregnant women in the Campinas region of the state of São Paulo and is far from ideal. Furthermore, to the best of our knowledge, few studies in the literature have evaluated the knowledge, attitude and practice of women regarding physical exercise and the reasons why the majority of women do not exercise.

Another limitation of the present study refers to the definition of what constitutes adequate knowledge. Defining adequate knowledge is a very complex matter, since it involves perceptions of right and wrong, level of access to different means of communication and each individual's life experience. For the purpose of this study, adequate knowledge was defined as the woman having heard about the performance of physical exercise during pregnancy and her being able to list which forms of exercise were appropriate and which should not be performed at this time. This interpretation was based on the recommendations of the American College of Obstetricians and Gynecologists (ACOG) on the practice of physical activity during pregnancy. We realize that this definition may have introduced an interpretation bias depending on the woman's access to the different levels of information. Nevertheless, since the population analyzed was reasonably homogenous, with similar socioeconomic conditions and dependent on the National Health Service for their healthcare requirements, we believe that this bias may be minimal.

Despite these limitations, we believe that these findings may collaborate towards improving the guidance given during prenatal care, serving as a subsidy for healthcare professionals, particularly those working in physical education, to enable them to improve their programs for pregnant women. Further interventions are necessary such as the possibility of designing and providing an appropriate program of physical exercise during pregnancy that could be made available and implemented at primary healthcare level in the clinics that provide care to these women. A study was carried out to evaluate a population of pregnant women in Campinas and results suggest that the practice of water-based physical activity is beneficial to pregnant women, although it was not associated with any increase in quality of life. Nevertheless, the study sample was very small, which may explain these findings [28].

We recommend that this population be given information on the benefits of the practice of simple, regular physical exercise such as supervised walking preceded by stretching exercises, for example, and encouraged to participate in this type of exercise. Costs would be insignificant and the return in terms of gestational well-being and health would be considerable.

Furthermore, from another viewpoint, these women may benefit from interventions at community level, aimed directly at the family, providing necessary social support and promoting and sustaining a healthy lifestyle. Some studies have shown that lack of information on changes for a healthier lifestyle could influence the behavior of this population. Identifying factors that affect beliefs and behaviors would objectively encourage a change in attitude [16].

For these measures to be implemented, healthcare managers need to be sensitized with respect to the actual benefits of the practice of physical exercise during pregnancy and to the possible control of certain pregnancy-related pathologies that are particularly common in populations with these characteristics. Without doubt, the population of pregnant women would be greatly benefitted by this service.

This report discusses the rationale behind the changes, and offers educational tools that may be employed to initiate behavioral change. We also propose exercise prescriptions for pregnant women. Armed with this information, the practitioner will be better equipped to counsel patients and incorporate a discussion on physical activity into prenatal visits.

## Conclusions

Our results suggest that women's knowledge concerning the practice of physical exercise during pregnancy is reasonable and their attitude is favorable; however, relatively few actually exercise during pregnancy.

## Competing interests

The authors declare that they have no competing interests.

## Authors' contributions

CPR and HM participated in all steps of the study, including research planning, data collection, analysis and writing the manuscripts. All authors gave suggestions, read the manuscript carefully, fully agreed on its content and approved its final version.

## References

[B1] Wolfe LA Davies GA Canadian guidelines for exercise in pregnancy Clin Obstet Gynecol 2003 46 2 488 95 10.1097/00003081-200306000-00027 12808398

[B2] ACOG Committee Obstetric Practice ACOG Committee Opinion. Number 267, January 2002: exercise during pregnancy and the postpartum period Obstet Gynecol 2002 99 171 173 10.1016/S0029-7844(01)01749-5 11777528

[B3] Barakat R Pelaez M Montejo R Exercise during pregnancy improves maternal health perception: a randomized controlled trial Am JObstet Gynecol 2011 204 xx xx 10.1016/j.ajog.2011.01.043 21354547

[B4] Katz J Exercícios aquáticos na gravidez 1999 1 São Paulo: Manole

[B5] Domingues MR Barros AJ Leisure-time physical activity during pregnancy in the 2004 Pelotas Birth Cohort Study Rev Saude Publica 2007 41 173 180 10.1590/S0034-89102007000200002 17420903

[B6] Clarke PE Gross H Women's behaviour, beliefs and information sources about physical exercise in pregnancy Midwifery 2004 20 133 141 10.1016/j.midw.2003.11.003 15177856

[B7] Krans EE Gearhart JG Dubbert PM Klar PM Miller AL Replogle WH Pregnant women's beliefs and influences regarding exercising during pregnancy J Miss State Med Assoc 2005 46 67 73 15822648

[B8] Gouveia R Martins S Sander AR Nascimento C Figueira J Valente S Correia S Rocha E Silva LJ [Pregnancy and physical exercise: myths, evidence and recommendations] Acta Med Port 2007 20 209 214 17868529

[B9] Duncombe D Wertheim EH Skouteris H Paxton SJ Kelly L Factors related to exercise over the course of pregnancy including women's beliefs about the safety of exercise during pregnancy Midwifery 2009 25 430 438 10.1016/j.midw.2007.03.002 18063253

[B10] Cioffi J Schimied V Dahlen H Mills A Thornton C Duff M Cummings J Kolt GS Physical activity in pregnancy: women's perceptions, practices and influencing factors J Midwifery Womens Health 2010 55 455 461 10.1016/j.jmwh.2009.12.003 20732667

[B11] Warwick DP Lininger AC Introduction in the sample survey, theory and practice 1975 New York: Mc Graw Hill 4 19

[B12] Pereira MA Rifas-Shiman SL Kleinman KP Rich-Edwards JW Peterson KE Gillman MW Predictors of change in physical activity during and after pregnancy: Project Viva Am J Prev Med 2007 32 312 319 10.1016/j.amepre.2006.12.017 17383562 PMC1894953

[B13] Evenson KR Moos MK Carrier K Siega-Riz AM Perceived barriers to physical activity among pregnant women Matern Child Health J 2009 13 364 375 10.1007/s10995-008-0359-8 18478322 PMC2657195

[B14] Lewallen LP Healthy behaviors and sources of health information among low-income pregnant women Public Health Nurs 2004 21 200 206 10.1111/j.0737-1209.2004.021302.x 15144364

[B15] Symons Downs D Ulbrecht JS Understanding exercise beliefs and behaviors in women with gestational diabetes mellitus Diabetes Care 2006 29 236 240 10.2337/diacare.29.02.06.dc05-1262 16443866

[B16] Thornton PL Kieffer EC Sabarría-Peña Y Odoms-Young A Willis SK Kim H Salinas MA Weight, diet, and physical activity-related beliefs and practices among pregnant and postpartum Latino women: the role of social support Matern Child Health J 2006 10 95 104 10.1007/s10995-005-0025-3 16534660

[B17] Rousham EK Clarke PE Gross H Significant changes in physical activity among pregnant women in the UK as assessed by accelerometry and self-reported activity Eur J Clin Nutr 2006 60 393 400 10.1038/sj.ejcn.1602329 16306930

[B18] Doran F O'brien AP A brief report of attitudes towards physical activity during pregnancy Health Promot J Austr 2007 18 155 158 17663652 10.1071/he07155

[B19] Mudd LM Nechuta S Pivarnik JM Paneth N Michigan Alliance for National Children's Study Factors associated with women's perceptions of physical activity safety during pregnancy Prev Med 2009 49 194 199 10.1016/j.ypmed.2009.06.004 19540874

[B20] Weir Z Bush J Robson SC McParlin C Rankin J Bell R Physical activity in pregnancy: a qualitative study of the beliefs of overweight and obese pregnant women BMC Pregnancy Childbirth 2010 28 10 18 10.1186/1471-2393-10-18 PMC2879230 20426815

[B21] Symons Downs D Hausenblas HA Women's exercise beliefs and behaviors during their pregnancy and postpartum J Midwifery Womens Health 2004 49 138 144 15010667 10.1016/j.jmwh.2003.11.009

[B22] Tavares JS Melo ASO Amorim MMR Barros VO Takito MY Benício MHD Cardoso MAA Padrão de atividade física entre gestantes atendidas pela estratégia da saúde da família de Campina Grande Rev Bras Epidemiol 2009 12 10 19

[B23] Schramm WF Stockbauer JW Hoffman HJ Exercise, employment, other daily activities and adverse pregnancy outcomes Am J Epidemiol 1996 43 211 218 10.1093/oxfordjournals.aje.a008731 8561154

[B24] Mottola MF Campbell MK Activity patterns during pregnancy Can J Appl Physiol 2003 28 642 653 10.1139/h03-049 12904639

[B25] Haakstad LA Voldner N Henriksen T Bø K Physical activity level and weight gain in a cohort of pregnant Norwegian women Acta Obstet Gynecol Scand 2007 86 559 564 10.1080/00016340601185301 17464584

[B26] Marquez DX Bustamante EE Bock BC Markenson G Tovar A Chasan-Taber L Perspectives of Latina and non-Latina white women on barriers and facilitators to exercise in pregnancy Women Health 2009 49 505 521 10.1080/03630240903427114 20013518 PMC2796193

[B27] Brasil Ministério da Saúde. Secretaria da Vigilância em Saúde, Decree #79 2008 http://HTTP//portal.saude.br/portal/arquivos/pdf/portaria_79_2008

[B28] Vallim AL Osis MJ Cecatti JG Baciuk EP Silveira C Cavalcante SR Water exercises and quality of life during pregnancy Reprod Health 2011 8 14 10.1186/1742-4755-8-14 21575243 PMC3113331

